# Optimizing Performance Monitoring of Value-Based Healthcare: The Role of Generic Patient Population Dashboards: Comment on "Reflections on Managing the Performance of Value-Based Healthcare: A Scoping Review"

**DOI:** 10.34172/ijhpm.9251

**Published:** 2025-12-20

**Authors:** Marieke Sijm-Eeken, Jurrien Burren, Céline van Lint

**Affiliations:** ^1^Department of Medical Informatics, Amsterdam Public Health Research Institute, Amsterdam UMC, University of Amsterdam, Amsterdam, The Netherlands.; ^2^Amsterdam UMC, University of Amsterdam, Amsterdam, The Netherlands.; ^3^Department of Quality and Patient Care, Erasmus MC University Medical Center, Rotterdam, The Netherlands.

**Keywords:** Value-Based Healthcare, Performance Management, Dashboard, Performance

## Abstract

Value-based healthcare (VBHC) provides a framework for enhancing health outcomes relative to costs, however its implementation depends on robust and inclusive performance monitoring systems. In their recent scoping review, van Elten et al examined the role of performance management in VBHC but adopted a narrow focus on patient-perceived outcomes. This commentary extends their findings by highlighting two key recommendations: expanding performance measurement beyond patient-perceived value to include provider experience, innovation, and transparency, and implementing generic patient population dashboards to streamline VBHC monitoring. Drawing on practical implementation experience at Dutch academic hospitals, we propose broadening literature search criteria to include cross-organizational studies and grey literature from established VBHC organizations. Furthermore, we outline criteria—identified through co-design sessions with healthcare professionals—for selecting generic indicators and designing dashboards that support efficient, organization-wide VBHC monitoring. These insights seek to enhance the theoretical and practical understanding of VBHC monitoring while promoting the advancement of transparent, value-driven healthcare systems.

## Introduction

 Value-based healthcare (VBHC) has emerged as a promising approach for optimizing healthcare organizations, grounded in principles such as organizing care around patient pathways for specific conditions.^1^ Like any management strategy, it is essential to monitor both the adoption and effectiveness of VBHC to identify opportunities for further improvement. In their scoping review, van Elten et al^2^ investigated the use of performance management systems within VBHC initiatives, aiming to inform future successful implementation. As they observed, there is a relative scarcity of studies detailing the benefits and successful applications of performance management in the context of VBHC.

 However, this does not imply such examples are nonexistent. While literature reviews such as those conducted by Van Elten et al^2^ are important, they are only a starting point. The purpose of this commentary is to reflect on and extend the work of Van Elten et al,^2^ thereby advancing the field’s understanding of VBHC monitoring.

## Methods

 The approach followed for assessing and reflecting on the scoping review reported by van Elten et al in the commentary, is to first review the definitions used by van Elten et al for VBHC performance and performance monitoring. Second, we reflect on the scope and depth of the review and on how to improve those. Next, we asses the results of van Elten and colleagues’ scoping review results and compare them to our experiences with challenges on VBHC monitoring in Dutch academic hospitals. We finish by presenting future recommendations for improving VBHC monitoring informed by recent developments in VBHC implementation, drawing on both established VBHC theory and our practical experience on implementing VBHC monitoring in leading Dutch university hospitals.

## Definitions of Value-Based Healthcare Performance and Performance Monitoring

 van Elten et al provides a succinct overview of VBHC, highlighting patient benefits, the transition from volume to value, and the importance of value across the care continuum. However, their definition does not capture several critical components of the VBHC framework.^1,3^ Notably, it omits value drivers such as provider experience,^1,3^ competition based on outcomes^1^ (and at broader levels, such as regional or national), incentives for innovation,^1^ and the transparency of performance data.^1^ We believe that a comprehensive understanding of VBHC must include these dimensions, and that any meaningful assessment of VBHC performance should reflect this broader perspective. Accordingly, we propose the following definitions:

 VBHC performance is defined as the extent to which a healthcare organization achieves improved patient outcomes relative to costs, while also fostering provider experience, encouraging innovation, promoting competition based on outcomes, and ensuring transparency of performance data across the entire care pathway.

 Based on this definition, VBHC performance monitoring is defined as the systematic, continuous measurement and evaluation of patient outcomes and experiences, costs, provider experience, innovation activities, competitive dynamics, and performance transparency, with the goal of informing and optimizing VBHC delivery within and across organizations.

## Opportunities to Broaden the Scope and Depth of the Review

 van Elten et al conducted their scoping review in accordance with the Preferred Reporting Items for Systematic Reviews and Meta-Analysis Protocols for Scoping. Building on this rigorous approach, we propose three suggestions to further enhance the scope and depth of the search strategy and selection criteria.

 First, incorporating additional keywords such as “PROMs” (patient-reported outcome measures), “PREMs” (patient-reported experience measures), “quality indicators” (registry indicators also used for performance management), “steering information” (management tools) and “population (health) management” (national and regional initiatives) could help capture a broader range of relevant literature. Many studies related to VBHC, such as that by Kendrick et al,^4^ do not explicitly use the term “value-based” in their titles or abstracts, despite being conceptually aligned with VBHC principles. PROMs and PREMs are commonly used indicators in value-based performance measurement and may serve as important proxies for identifying such work.

 Second, the review focused exclusively on studies addressing aspects of the internal healthcare organization, excluding 199 articles (15.7% of initially identified records) that for instance addressed inter-organizational or cross-sectoral procurement. Given that a core principle of VBHC is to optimize outcomes across the entire care continuum,^1^ these excluded studies may offer valuable insights into current practices in performance management systems that span organizational boundaries.

 Third, in addition to peer-reviewed literature, practical frameworks and best practices developed by leading organizations such as the International Consortium for Health Outcomes Measurement,^5^ the European Health Management Association,^6^ and the Linnean Initiative^7^ could provide meaningful contributions. These sources offer implementation guides, metrics, and tools that are actively used to support VBHC performance measurement in practice and could have enriched the review’s findings concerning the extent to which performance management systems support VBHC implementation.

## Reflection on Current State of Value-Based Healthcare Performance Measurement

 In this paragraph, we reflect on the scoping review results presented by van Elten et al, specifically on the completeness of findings and how they align with our experiences from implementing VBHC in Dutch university hospitals. While a key principle of VBHC is to consider the full care pathway across different healthcare providers rather than care provided within one or a few organizations,^1^ none of the articles identified in the review by van Elten et al considered the care pathway.^2^ We share their observation that the VBHC concept of cross-organizational cooperation might still be in an early stage. In addition, we consider measuring VBHC performance within a single healthcare institution to be an important step towards full-cycle performance measurement.

 In the introduction of their paper, van Elten et al underscore the limited use of patient-centered measurements and the prevailing emphasis on volume rather than value in healthcare systems.^2^ This characterization does not reflect the practices we experienced at two Dutch university hospitals in which we worked on VBHC projects. In these settings, a broad array of PROMs and PREMs are routinely used to monitor health outcomes.^8-10^ In addition, data is collected systematically on clinical outcomes (eg, complication rates, survival rates), internal hospital metrics (eg, bed availability), process indicators (eg, adherence to clinical pathways, patient involvement), and financial performance (eg, cost per patient day).

 While these practices illustrate that VBHC can be effectively implemented, several challenges persist. A major obstacle is not the lack of data—many relevant indicators are already available in existing information systems—but rather the substantial time and effort required to extract, process, and report this data.^8,9^ This is particularly demanding in settings involving multiple medical disciplines, each with their own VBHC performance measurement requirements and distinct sets of indicators. As the scope of VBHC expands, the number of indicators continues to grow, further increasing the burden of performance measurement and straining resources over time.

 Additionally, while current information systems offer various capabilities for VBHC measurement, certain patient-reported data are collected anonymously for privacy reasons and therefore cannot be linked to specific care pathways.

## Future Directions for Value-Based Healthcare Performance Measurement

 To address current challenges in VBHC performance measurement and to deliver improved outcomes for patients, further development of the approach used for monitoring is essential. A key priority for future improvement is the streamlining and standardization of VBHC performance monitoring to reduce the associated administrative burden and to enable effective organization-wide VBHC management. One promising strategy for achieving greater efficiency and standardization is the use of generic quality indicators that can be applied across multiple medical disciplines.^8^ This approach could reduce the overall number of indicators required and, in turn, lessen the administrative workload.

 The selection of appropriate generic indicators should be guided by two key considerations: (1) the availability of the required data within existing information systems, and (2) the preferences and practical needs of the healthcare professionals involved in both care delivery and management. A co-design methodology, involving representatives from both senior management (eg, directors, managers, and department heads) and healthcare providers (eg, medical specialists and nursing consultants), may offer an effective means of identifying a relevant and feasible set of generic, population-level indicators.

 Inspired by Mulhall et al,^11^ who described the successful implementation of a co-designed dashboard in Canadian long-term care homes, we applied a similar co-design approach to identify preferred generic indicators and dashboard requirements in a case study at a Dutch university hospital. Table 1 summarizes the identified generic indicators and corresponding reporting dashboard features, based on insights gathered from four co-design sessions with fourteen healthcare professionals.

**Table 1:** Preferred Requirements for a Generic Value-Based Healthcare Population Dashboard

**Requirements**	**Session I**	**Session II**	**Session III**	**Session IV**
Population demographic indicators				
Demographic data about the population	X	X	X	X
Accessibility of care	X		X	
Percentage of chronically illness	X			
Process indicators				
Number of outpatient visits (vs home consults)	X	X	X	X
Response rate of PROM’s	X	X	X	X
Therapy adherence		X	X	X
Waiting list		X	X	X
Referral numbers/flow	X	X		X
Medication	X		X	
Opening/discussion rate of PROM’s		X		X
Number of no-shows			X	
Staffing			X	
Clinical outcome indicators				
Diagnose defined clinical outcomes	X	X	X	X
Complications	X	X		X
Patient reported indicators				
Patient satisfaction scores	X	X	X	X
Quality of Life scores	X	X	X	X
Experienced pain scores		X	X	X
Mortality	X			X
Mental health scores			X	X
Mobility scores				X
Non-clinical outcome indicators				
Length of stay	X	X	X	
Insights into cost drivers	X		X	
Readmission	X	X		
Cost related indicators				
Number of requests diagnostics	X		X	
Average cost per patient	X			
Staffing			X	
Filtering				
Filtering for gender	X	X	X	X
Filtering for age	X	X	X	X
Filtering for diagnosis	X	X		X
Filtering for specialty	X	X		X
Filtering for pre/post intervention		X		X
Filtering for clinician			X	X
Other functional requirements				
Information button for more background information	X		X	
Hoover for more detail	X	X		
Possibility to export data	X			
Smartphone-view possible	X			

Abbreviation: PROM, patient-reported outcome measure.

 During the co-design sessions, a total of 28 requirements were identified. As illustrated in Table 1, the requirements are divided into eight categories. The filter requirements presented in category seven offers the possibility of zooming in to a subset of a patient population. Diagnose defined clinical outcomes, patient satisfaction scores and quality of life scores were identified as essential indicators in all co-design sessions. Similarly, the inclusion of filters for gender and age was consistently mentioned as a preferred feature of the reporting dashboard across all sessions.

Table 2 presents an overview of the participating healthcare professionals and their self-reported objectives for using VBHC performance dashboards, specifically in relation to a generic VBHC population dashboard. In contrast to the findings of van Elten et al^2^— who observed a lack of alignment between VBHC performance measurement and strategic organizational goals—the goals reported in this case study align with the hospital’s five-year strategic plans.

**Table 2:** Participants of the Co-design Sessions and Their Objectives for a Generic Value-Based Healthcare Population Dashboard

**Function**	**N**	**Session**	**Goals (Preceded by Participant Identifier)**
Theme director	2	I and IV	TD1: Selection of which patients would be suitable for treatment outside the hospital or from home. TD2: Making information at a higher level of abstraction available. Achieve combination with clinical interventions and clinical outcomes.
Quality advisor Theme director	2	I and IV	QA1: Based on predefined indicators, see how a department/a theme/the hospital is doing in terms of Appropriate Care. QA2: Management information that can be used by the floor and best practice information.
Department head	1	IV	DH1: Provide insight into relevant VBHC parameters for target population. Focus on steering information that is for higher level. On selected designs mirror for subgroups if applicable.
VBHC delegate Department head	2	I and IV	VD1: Care for patients/intervening, space for changing outpatient structures (regulatory appointments versus targeted appointments). VD2: How to set up care, visibility, questionnaire completion/discussion rate.
Medical specialist	2	II and III	MS1: Completion/discussion rate, variation over time, variation between target group (liver transplant) variation between organ groups. MS2: Course of frequency completing questionnaires, adjust frequency of release per questionnaire.
Nurse practitioner	3	II	NP1: Trends and future predictions on disease course. NP2: What care is needed at what time, what is an improvement for the patient? How does the patient perceive his care? NP3: Trends.
Nurse consultant	2	III	NC1: Questionnaires modification to the population. NC2: Adapting questionnaire to patients.

Abbreviations: VBHC, value-based healthcare; TD, theme director; QA, quality advisor; DH, department head; MS, medical specialist; NP, nurse practitioner; NC, nurse consultant; VD, VBHC delegate.

 The requirements outlined above were translated into a functional prototype within one month. This case study illustrates the feasibility of developing a generic VBHC population dashboard that can support broader implementation efforts and facilitate systematic VBHC performance monitoring. Nonetheless, we concur with van Elten et al^2^ that the implementation of VBHC initiatives—such as performance management tools—remains a complex endeavor.

 Our experience combining a bottom-up development process with the involvement of top-down stakeholders through co-design suggests a promising approach for successful implementation and adoption of VBHC. In the Dutch healthcare context, the VBHC principle of fostering competition based on outcomes requires further consideration, as it is currently only partially realized. Another aspect needing improvement is the transparency of performance data across the entire care pathway.

 Based on our working definition of VBHC performance monitoring, the review by van Elten et al, and our practical experience with VBHC implementations, we conclude that systematic, continuous measurement and evaluation of various VBHC indicators have become common practice at some hospitals such as those where we are involved in. However, the implementation of metrics specifically targeting innovation activities and competitive dynamics remains limited. Furthermore, there is a need to enhance performance transparency by sharing and publishing VBHC performance data more broadly.

 Increasing transparency may be challenged by concerns over potential negative consequences in settings where allocation of care is linked to performance quality. To address this, healthcare policies should support ongoing refinement of care delivery by mitigating such concerns and fostering a culture in which performance transparency is standard practice.

 Sharing performance measures with the outside world could enable the type of competition that Porter and Teisberg^1^ described as a critical step on the path towards VBHCwhere health improvement and service outcomes play a central role.

## Conclusions

 The work of van Elten et al^2^ provides a valuable foundation for exploring the integration of performance management systems within VBHC. Building on their review, we emphasize the importance of broadening the scope of performance indicators and integrating cross-disciplinary, co-designed dashboards. While promising progress has been made in institutions like the university hospitals where we are involved in, further efforts are needed to incorporate innovation metrics, foster value-based competition, and ensure transparency of outcomes. Enhancing performance monitoring in this way is essential to achieving the full potential of VBHC and enabling a more sustainable and patient-centered healthcare system.

## Disclosure of artificial intelligence (AI) use

 Not applicable.

## Ethical issues

 Not applicable.

## Conflicts of interest

 Authors declare that they have no conflicts of interest.
